# Application of propolis extract, nanovitamin C and nanovitamin E to prevent alveolar osteitis after impacted lower third molar surgery. A randomized, double-blind, split-mouth, pilot study

**DOI:** 10.4317/medoral.23915

**Published:** 2021-02-20

**Authors:** José González-Serrano, Rosa María López-Pintor, Roberto Cecilia-Murga, Jesús Torres, Gonzalo Hernández, Juan López-Quiles

**Affiliations:** 1 PhD Student. Department of Dental Clinical Specialities. ORALMED research group. School of Dentistry, Complutense University of Madrid, Spain; 2 Department of Dental Clinical Specialities. ORALMED research group. School of Dentistry, Complutense University of Madrid, Spain; 3 Department of Dental Clinical Specialities. School of Dentistry, Complutense University of Madrid, Spain

## Abstract

**Background:**

Propolis has anti-inflammatory, analgesic and healing properties. The purpose of this study was to determine whether a gel containing 2% of propolis extract, 0.2% of ascorbic acid and 0.2% of tocopherol acetate is effective in preventing surgical complications related to impacted lower third molar extractions.

**Material and Methods:**

A randomized, double-blind, split-mouth study was performed. Fifteen patients were recruited who needed bilateral impacted lower third molar extractions with a similar surgical difficulty. A test or placebo gel was administered randomly inside post-extraction sockets. Each patient was instructed to apply the gel 3 times/day in the surgical wound for a week. After a month, the contralateral third molar was extracted, and the opposite gel applied. The following parameters were diagnosed/evaluated and then recorded: alveolar osteitis following Blum’s criteria, swelling and trismus at day one, two, three and seven post-intervention, wound healing at day 7 post-intervention, and postoperative pain using a visual analog scale, as well as, the number of analgesic pill intake.

**Results:**

A total of twenty-six surgical procedures were performed in 13 patients (mean age 20.67±2 years). Alveolar osteitis was reported in 3 patients from the placebo group (23.1%) and none in the test group (0%) (*p*=0.25). No statistically significant differences were reported in swelling, trismus, wound healing or analgesic pill consumption between two groups. But statistically lower postoperative pain during the 7 days after surgical extractions was found according to visual analog scale in test group compared to the placebo group (*p*=0.007). No side effects were reported.

**Conclusions:**

The application of this gel may be effective in preventing alveolitis and thus reducing postoperative pain after impacted third molar extractions. More randomized clinical trials with larger sample are needed to confirm these results.

** Key words:**Propolis, nanovitamin, third molar surgery, oral surgery, alveolar osteitis.

## Introduction

Lower third molars (3Ms) are the most frequently impacted teeth. In many cases the presence of impacted 3Ms is related to some problems such as pericoronitis, development of tumors, cysts or reabsorption and caries of the adjacent molars (1-3). The American Association of Oral and Maxillofacial Surgeons recommends the extraction of asymptomatic 3Ms based on clinical studies that investigated the occurrence and progression of such lesions related to impacted teeth. For these reasons, lower 3Ms extraction are one of the most common procedures in oral and maxillofacial surgery (4).

However, its extraction leads to a series of postoperative complications such as alveolar osteitis (AO), infections, dysesthesia, bleeding, swelling, pain or deficient wound healing (5). AO is one of the most common postoperative complications with a prevalence that ranges from 1 to 37.5% (6). Insufficient blood supply of the socket, traumatic extractions, heavy sucking or spitting postoperatively, bacterial invasion, and the consequent loss of the clot have been proposed as its causes (7). The onset of AO is as an urgent dental problem that implies multiple follow-up visits and patients discomfort (8).

Different pharmacological products including antibiotics, anti-inflamatory agents, antiseptics, antifibrinolytics and recently plasma rich in growth factors have been used with contradictory results (9,10). Nonetheless, the use of systemic antibiotics does not eliminate the risk of appearance of AO (11) and may develop bacterial resistances (12). Also the use of antiseptics, anti-inflammatory and analgesics drugs does not completely reduce swelling and pain (13). Consequently, local interventions have been used to minimize these complications, yet a Cochrane systematic review concluded that there was no evidence supporting any local procedure that prevent AO (14). So, research of new treatments capable of reducing the incidence of AO is necessary.

The use of propolis in Medicine and Dentistry is mainly due to its anti-inflammatory, antibacterial, antifungal, analgesic and healing properties (15). Propolis has been used as a bone-grafting substitute for the management of periodontal defects (16) and has shown to stimulate bone regeneration through inhibiting osteoclastic activity (17). A previous study evaluated the oral microflora using 3% ethanolic solution of propolis extract after the extraction of 3Ms, obtaining a reduction against facultative anaerobic oral microorganisms (18). Moreover, Cosola *et al*. (19) evaluated a gel containing propolis extract, nanovitamin C and nanovitamin E and found a lower bacterial colonization on suture threads after surgical procedures in the oral cavity when compared to 0.2% clorhexidine gel application, but no pain or AO was assessed. To our knowledge, there are no studies that have evaluated the application of propolis extract, nanovitamin C and nanovitamin E gel (NBF gingival gel, Sungwon pharmaceutical co., Ltd. South Korea) to prevent complications related to the extraction of the lower 3Ms.

The aim of this study was to perform the first clinical trial to assess the effectiveness and safety of this gel in controlling post-interventional complications in patients undergoing surgical extraction of mandibular 3Ms.

## Material and Methods

This study followed the guidelines established by the CONSORT (Consolidated Standards of Reporting Trials) checklist (http://www.consort-statement.org/).

- Study design

This study was performed in a single-centre (Complutense University of Madrid). It was a double-blind, randomized and split-mouth clinical trial. Each patient underwent extraction of the two lower wisdom teeth in two different surgeries a month apart between them, and randomly received one type of treatment each time (test or placebo). Both patients and researchers did not know what treatment was being used.

- Ethics

The Ethics Committee at Hospital Clínico San Carlos of Madrid (Spain) approved the study protocol in accordance with Helsinki Declaration (Protocol No. 16/314-P). The study protocol was registered at clinicaltrials.gov (Number: NCT03641482). Prior to inclusion, the study was explained to the potential participants, who also received a written informed consent that they had to sign several days before the surgery, thus agreeing to participate in the study.

- Participants

Patients who attended the Oral Surgery and Implantology Master Programme Clinic, at the School of Dentistry at Complutense University of Madrid (Spain), between September 2016 and July 2017, were recruited.

The inclusion criteria were: (a) cooperative adult patients able to fulfill the study protocol, (b) needing surgical extractions of both lower 3Ms, (c) with moderate difficulty (scores between 5 and 6) according to Pederson scale (20).

The exclusion criteria were: (a) refuse to participate in the study, (b) failure to attend 24, 48, and 72 hours, as well as, 7 days post-surgical appointment visits, (c) smokers, (d) systemic diseases as diabetes mellitus or immunosuppresion, (e) patients taking oral contraceptives, (f) patients who had taken local or systemic antibiotics less than 3 months ago, anti-inflammatory or anticoagulant medication in the previous 4 weeks, (g) patients who required antibiotic prophylaxis, (h) pregnant or breastfeeding women, (i) patients with periodontitis in active phase and/or (j) with history of allergies to local anaesthetics, antibiotics, AINEs, test gel (TG) or placebo gel (PBG) components.

- Randomization and blinding

The company Bio Nature Essence S.L., trading company of propolis extract, nanovitamin C and nanovitamin E gel in Spain, provided the TG and PBG. Both preparations were of a gel consistency, and were contained in identical tubes (30g). PBG was similar to TG in color, flavor and density. The components present in both the PBG and TG were: Sodium-Monofluorophosphate, Silicon Dioxide, Glycerin, D-sorbitol, Polyethylene glycol, Sodium Carboxymethylcellulose, Xylitol, Sterol Glycoside, Peppermint Oil, L-Menthol, Methyl Hydroxybenzoate and Deionized Water. Propolis Extract (2%), Ascorbic Acid (0.2%) and Tocopherol Acetate (0.2%) were only present in TG. E155/151 coloring was only present in PBG to simulate the brown color of the propolis extract present in TG.

The use of TG and PBG in each patient was determined with a random number generator by the company Bionature Essence S.L. TG and PBG tubes were numbered consecutively from 1A to 15A and from 1B to 15B. Hence, A tubes would not always be PBG or B tubes would not always be TG, and vice versa.

Patients who met the eligibility criteria were randomized to the type of treatment (TG or PBG) and to the side of the first surgery (right or left). The side of the first surgery was determined by tossing a coin. Patients received the corresponding gel tube A (TG or PBG) after the first surgery. One month later, the remaining wisdom tooth was extracted and gel tube B with the opposite gel was applied.

The patients, the oral surgeon (José González-Serrano) (JGS) and the researcher who collected the data (Roberto Cecilia-Murga) (RCM) ignored the gel that they were using. The randomization code was revealed after all patients finished the trial and before analyzing the data, performing a double-blind study.

- Interventions and instructions to patients

All 3Ms surgical interventions were performed by the same oral surgeon (JGS) with extensive experience in these types of procedures. An ortopantomography was used to classify the extraction difficulty according to Pederson scale (20), and only those presenting moderate difficulty (scores between 5 and 6) were selected. All surgical procedures were done under local anaesthesia with 4% articaine and 1:100,000 epinephrine. After a lineal incision from mesial of the lower first molar with a distal extension to the mandibular ramus was performed, a mucoperiosteal flap was raised and ostectomy was done using a No. 8 tungsten carbide bur mounted on a handpiece. When necessary, the molar was sectioned with the same bur. An elevator was used to complete the extraction procedure.

Once the molar was extracted, the socket was cleaned with saline solution, and gel from tube A was introduced into the socket. Afterwards, the flap was sutured with two isolated stitches using 3-0 non-resorbable silk thread (Ergon Sutramed S.p.A., Magliano del Marsi, AQ, 67062, Italy). All the patients were prescribed amoxicillin 750mg 3times/day for 7 days, ibuprofen 600mg 3times/day for 3 days, and magnesium metamizole 575mg only when necessary. Gel tubes A were given to the patients. They were instructed to apply it in the surgical wound 3 times per day for 7 days after brushing their teeth. Previously, they had to dry the area of the wound with gauze. Suture was removed after 7 days.

The same oral surgeon (JGS) performed the contralateral extraction one month after the first intervention and the opposite gel (gel B) was applied. Therefore, each subject received both gels (TG and PBG) in a split-mouth design manner.

- Clinical examination

A non-operating investigator (RCM) recorded pre-operative and post-operative measurements (AO, swelling, trismus and wound healing). RCM was blinded to the intervention used.

AO was assessed after 24, 48 and 72 hours of the intervention. AO was diagnosed following Blum’s criteria (7). Extraoral swelling was measured with a 3-0 silk suture put between tragus and pogonium, following the maximal convexity of the cheek, and measured against a rule. Trismus were evaluated using a Vernier gauge to measure the interincisal distance between the right upper and lower central incisors (21). A first measurement was made before surgery, which was compared with the measurements taken at 24, 48, 72 hours and 7 days after the extraction.

After 7 days, when the suture was removed, wound healing was classified as ‘good’, ‘accepTable’ or ‘bad’ according to Madrazo-Jiménez *et al*. scale (22), which consider 3 characteristics: wound edges, color of the mucosa and wound closure. ‘Good’ is considered when wound edges are aesthetic, clean and with good opposing edges; the color of the mucosa is identical to the surrounding area; and wound closure is complete or without dehiscence. ‘AccepTable’ is considered when wound edges are slightly irregular, with light bleeding or erythema; the color of the mucosa is similar to the surrounding area; and wound closure presents 1-2mm dehiscence. Finally, ‘bad’ is considered when wound edges are irregular, with moderate or heavy bleeding, exudate, pus, foul odor and/or signs of infection; the color of the mucosa is erythematous and wound closure presents a dehiscence >2mm, open wound, keloid formation or unaesthetic closure.

The patients recorded postoperative pain using a 10-cm horizontal visual analog scale (VAS), ranging from 0 (“no pain”) to 10 (“the worst pain imaginable”) at 9 pm for 7 consecutive days. The patients also recorded the number of rescue analgesic pill consumption (magnesium metamizole 575mg) for the first 3 days post-intervention.

- Sample size and statistical analysis

We calculated the sample size according to the data from the study by Haraji and Rakhshan (20) using clorhexidine gel 0.2% versus placebo in the prevention of AO after lower 3Ms extractions. A 35.6% of patients receiving placebo of this study suffered AO after lower 3Ms surgery. In our study, we estimated that less than 4% of the test group would suffer AO. A sample size of 13 subjects in each study group was estimated to be required to obtain 80% power to detect this effect as statistically significant (=0.10).

SPSS software for Windows version 25.0 (SPSS Inc, Chicago, IL, USA) was used for statistical analysis. Statistical analysis comprised basic descriptive statistics. Changes between baseline and measurements taken at 24, 48, 72 hours and 7 days after the extractions were calculated. Mean VAS scores during the 7 days after surgical interventions and mean consumption of analgesic pills were obtained. Shapiro Wilk goodness-of-fit test were used to determine the normal distribution of the quantitative variables. Due to the split-mouth study design, comparison between the PBG and TG sides was performed using Wilcoxon signed-rank test. To assess possible differences among dichotomous variables McNemar’s test was used. We used Friedman's test to analyze repeated intragroup measurements. Significance level was set at *p* 0.05.

## Results

- Participant flow

**Figure 1:**
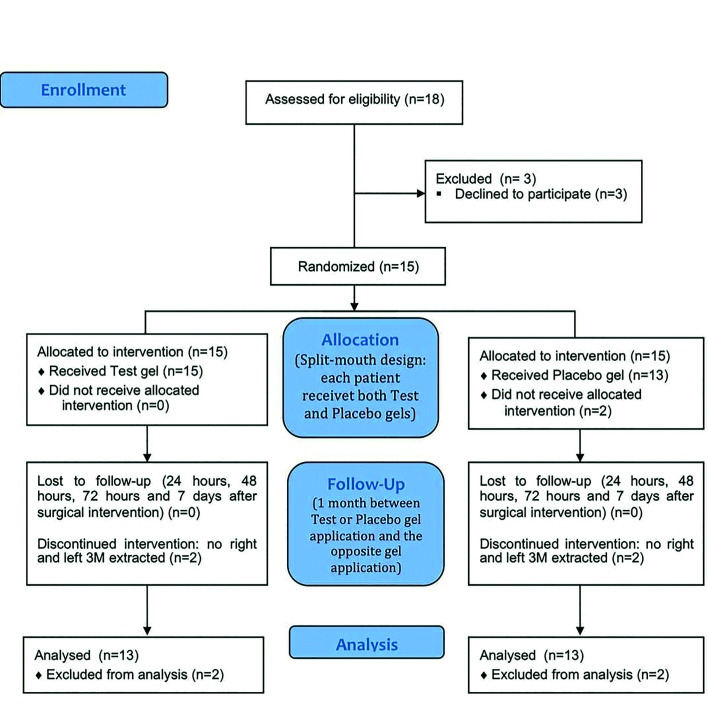
Consort Flow diagram on subject enrollment, allocation, follow-up and analysis.

Eighteen patients were recruited to participate in this study. After recruitment, 3 patients refused to participate for work reasons and were withdrawn before randomization. Therefore, fifteen patients were included in this study. Thirteen finally completed the study (10 women and 3 men; mean age 21.15±2.03 years). Two patients did not wish to perform the second left or right wisdom tooth extraction and were excluded post-randomisation (Fig. 1).

- Baseline data

After a split-mouth design, the variables age and gender were the same for test and placebo groups. The molar position and surgical difficulty of the sample are shown in Table 1.

- Outcomes

AO was reported in 3 out of 13 sockets (23.10%) in PBG group and in none (0%) of the TG group (*p*=0.25).

**Figure 2:**
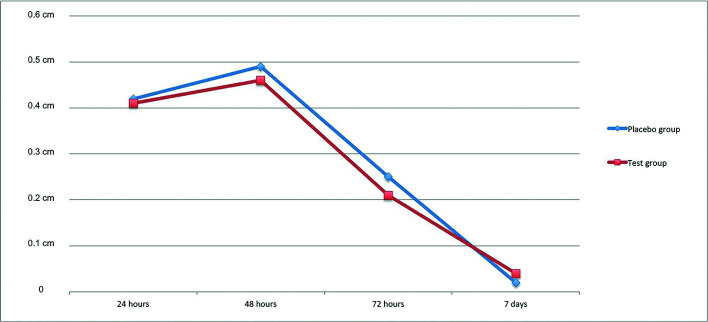
Increase of inflammation with regard to the first day in Test and Placebo groups 24, 48, 72 hours and 7 days post-surgical intervention.

Mean swelling increase with regard to preoperative status was 0.42±0.24cm and 0.41±0.28cm (*p*=0.92) in the first postoperative day, 0.49±0.37cm and 0.46±0.38cm (*p*=0.85) in the second postoperative day, 0.25±0.3cm and 0.21±0.3cm (*p*=0.59) in the third postoperative day, and 0.02±0.05cm and 0.04±0.14cm (*p*=0.65) in the seventh postoperative day in PBG and TG, respectively (Fig. 2). Intragroup changes in swelling at all four assessment points were significant in both groups (*p*=0.0001).

Mouth opening capacity (trismus) was reduced in 1.58±1.29cm (30.68%) and 1.98±0.85cm (38.82%) (*p*=0.27) in the first postoperative day, 1.37±1.22cm (26.6%) and 1.62±0.89cm (31.76%) (*p*=0.78) in the second postoperative day, 1.05±1.11cm (20.39%) and 1.08±0.82cm (20.2%) (*p*=0.75) in the third postoperative day, and 0.49±0.89cm (9.51%) and 0.51±0.49cm (10%) (*p*=0.44) in the seventh postoperative day for PBG and TG, respectively. Intragroup changes in mouth opening capacity at all four assessment points were significant in PBG (*p*=0.001) and TG (*p*=0.0001).

**Figure 3:**
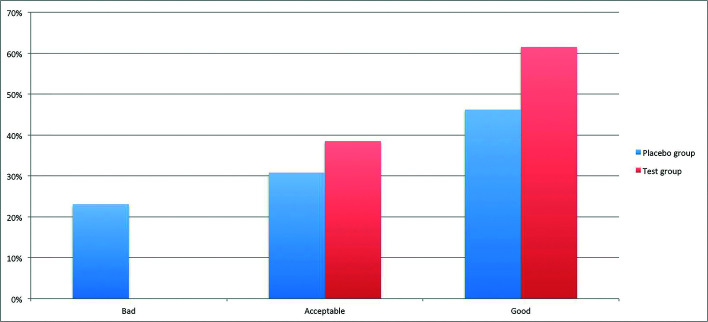
Wound healing proportion in Test and Placebo groups at seventh postoperative day classified according to Madrazo-Jiménez *et al*. scale.

Wound healing was considered ‘bad’ in 23.1% and 0%, ‘accepTable’ in 30.8% and 38.5%, and ‘good’ in 46.2% and 61.5% of PBG and TG sockets at the seventh postoperative day (*p*=0.16), respectively (Fig. 3).

The mean VAS scores during the 7 days after surgical intervention were statistically lower in TG (2.86±1.74) when compared to PBG (3.92±1.65) (*p*=0.007). A mean of 0.63±0.57 analgesic pills consumption was recorded in PBG and 0.38±0.39 in TG during the first three days after 3Ms extractions (*p*=0.16).

- Harms

No adverse reactions or discomfort were reported with either TG or PBG application.

**Table 1:**
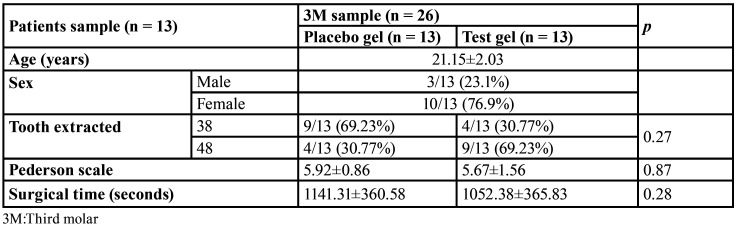
Sample and surgical variables.

## Discussion

The propolis properties may prevent AO after 3Ms extraction. The results of the present study show that the presence of AO was lower in TG than in PBG, however no statistically significant differences were observed. It may be explained because the application of TG in suture threads is related to a lower bacterial colonization compared with the use of chlorhexidine (19) and also the antioxidant effect of nanovitamin C and E of TG used in the present study could have added a positive effect in preventing AO (23).

There are no previous studies about the use of TG after the extraction of lower 3Ms. It is therefore not possible to compare our results with other similar studies using this product. Popovska *et al*. (24) published a case report of a patient that obtained the complete healing of oral lesions of erosive lichen planus by applying TG gel 3 times a day for 4 weeks. Additionally, in a randomized controlled study, Dednath *et al*. (25) obtained statistically significant differences at 3 months in probing pocket depth and clinical attachment level when TG gel was applied together with scaling and root planning, compared to a control group where scaling and root planning was only performed. Also, in a similar study, Giammarinaro *et al*. (23) found significantly better improvement of the oxidative status of saliva using TG when compared to chlorhexidine. Therefore, more scientific research is required to confirm the benefits of TG as antibacterial, wound healing, anti-inflammatory, analgesic, or probiotic agent to treat oral diseases.

Nonetheless, several local treatments have been used to prevent post-surgical complications after 3Ms extractions, with chlorhexidine being the most studied (14). These treatments have been applied intra-alveolar, topically, in rinses or in combination with each other. A meta-analysis (26) evaluating intra-alveolar 0.2% chlorhexidine gel application for the prevention of AO after mandibular 3Ms extractions showed AO rates of 0-23% and 5-35.6% in test and control groups, respectively. However, only 3 of the 11 studies selected in the meta-analysis (26) had a split-mouth design, which limits the control of variables such as oral hygiene. Moreover, 6 of the 11 studies included smokers (26), which may have altered AO rates (27). In our study, we excluded smokers and we applied intra-alveolar and topical TG or PBG with a split-mouth design, obtaining AO rates of 0% and 23.10% in TG and PBG, respectively. Therefore, it seems that TG gel achieved such good AO rates as chlorhexidine. Consequently, TG gel may be an alternative to clorhexidine, since there are some complications and side effects related to chlorhexidine application, such as mild contact dermatitis and anaphylaxis, dysgeusia, as well as, bacterial resistances with its prolonged use (28). Chlorhexidine has also been used in combination with chitosan, which can cause allergic reactions to people who are allergic to shellfish (22,29).

Regarding other complications related to the extraction of lower 3Ms, the present study found no statistically significant differences between two groups in mouth opening, swelling and analgesic pills consumption, but statistically lower postoperative pain according to VAS was found during the 7 days after surgical extractions in TG compared to PBG. Concerning the use of chlorhexidine, Jesudasan *et al*. (30) obtained that 0.2% chlorhexidine gel decreased pain, inflammation and showed better wound healing when comparing to a control group. Similarly, López-López *et al*. (29) compared the efficacy of 0.2% chlorhexidine, dexpanthenol, allantoin and chitosan gel versus bicarbonate oral rinse and found statistically significant reduction in pain intensity at day 7, swelling and lower analgesic pills consumption in the test group. However, Madrazo-Jiménez *et al*. (22) evaluated the same gel with 0.2% chlorhexidine, dexpanthenol, allantoin and chitosan and found that there were no statistically significant differences in facial swelling, trismus and postoperative pain between study and placebo groups. Nonetheless, they reported statistically significant differences in wound healing on day 7. They observed that 80% of test group presented ‘good’ healing vs. 28% in placebo group. In the present study, we found that wound healing in the seventh postoperative day was ‘good’ in 61.5% and 46.2% of the sockets in TG and PBG, respectively, although no statistically significant differences between two groups were observed.

The main limitation of this pilot study is the sample obtained, as it was difficult to obtain patients with two impacted lower 3Ms with similar surgical difficulty and to make them come to our dental clinic at 24, 48, 72 hours and 7 days after the surgery. Another limitation is the intake of antibiotics during the postoperative period. However, in the postgraduate program where the study was performed, it corresponds to the daily clinical practice.

In conclusion, this study shows that a gel containing propolis extract, nanovitamin C and nanovitamin E is safe, reduces postoperative pain and may also reduce AO after impacted lower 3Ms extractions. It is necessary to perform more randomized controlled clinical trials with larger samples to confirm these positive results.
